# Challenges in Translating Germinal Stem Cell Research and Therapy

**DOI:** 10.1155/2016/4687378

**Published:** 2015-11-09

**Authors:** Irma Virant-Klun, Hong-Thuy Bui, Mariusz Z. Ratajczak

**Affiliations:** ^1^Department of Obstetrics and Gynaecology, University Medical Centre Ljubljana, SI-1000 Ljubljana, Slovenia; ^2^Department of Biotechnology, School of Biotechnology, International University, Vietnam National University, Ho Chi Minh City 70000, Vietnam; ^3^Stem Cell Institute, James Graham Brown Cancer Center, University of Louisville, Louisville, KY 40202, USA

The field of stem cells research is one of the most exciting and fast developing fields with a great potential to heal different degenerative diseases in regenerative medicine, to better understand cancer and to improve quality of human life. In spite of great enthusiasm and effort, there are several general obstacles and questions about stem cells, which still need to be resolved to enable safe and efficient treatment of degenerative diseases in the future. There are more interesting types of stem cells in humans, as also evidenced by thirteen manuscripts published in this special issue.

Very small embryonic-like stem cells (VSELs) represent one of the most interesting types of stem cells in humans. These small stem cells are suggested to originate in epiblast of an embryo and to persist in a quiescent state in adult human tissues and organs (e.g., bone marrow) from embryonic period of life. They are mobilized into peripheral blood to regenerate the damaged area. It is not excluded that they are involved in tumor formation at inappropriate condition. They have been proven to be multipotent with a degree of pluripotency and able to differentiate into different cell types of all three germ layers. In the manuscript by H. Sovalat et al. it is shown that VSELs have been present in the peripheral blood of healthy volunteers in the same numbers regardless of their age, ranging from 20 to 70 years. Interestingly, endurance exercise such as long-distance running mobilizes VSELs into peripheral blood and increases their number in bone marrow in favor of regeneration, as found by K. Marycz et al. The VSELs have also been identified in bone marrow of other mammalian species such as rat (A. Labedz-Maslowska et al.). Moreover, the VSELs have been proposed by D. Bhartiya et al. to exist in adult mammalian ovaries and to participate in primordial follicle assembly and postnatal oogenesis under the regulation of follicle-stimulating hormone (FSH). This idea has been experimentally supported by M. Zbucka-Kretowska et al. who evidenced the effective release of VSELs into peripheral blood of women treated by FSH hormonal therapy to retrieve the oocytes in the in vitro fertilization program.

The VSELs are related to germinal lineage, especially primordial germ cells (PGCs), and it is not excluded that they are related to germinal stem cells (GSCs) and may be even their progenitors. The PGCs, precursors of gametes, can be generated from stem cells, thereafter, result in birth of mouse pups, and represent an exciting new tool to treat infertility in the future, as reviewed by A. Nikolic et al. The female GSCs, namely, oogonial stem cells, can be efficiently isolated from adult mouse ovaries by a new method provided by Z. Lu et al. and might be also applied to human ovaries. B. Fereydouni et al. have demonstrated the development of oocyte-like cells in long-term cell cultures of neonatal marmoset monkey ovary expressing several genes related to the germinal lineage. The ovarian GSCs are very interesting to regenerate the nonfunctional ovaries although also some other types of cells such as human amniotic epithelial cells can express a beneficial paracrine effect on function of chemotherapy-treated ovaries in a mouse model (X. Yao et al.). The GSCs can also be retrieved from adult human testicles but are heterogeneous and, along with genes related to the germinal lineage, express more or less pluripotency, as reported by S. Conrad et al. In a mouse model, derivation of embryonic-like stem cells from spermatogonial stem cells (SSCs) depends on animal age (up to 7 weeks of age) and special time window in long-term SSC cultures (H. Azizi et al.). Not only reproductive tissues but also parthenogenetic embryos may represent an interesting source of pluripotent stem cells after activation of nonfertilized oocytes from the in vitro fertilization program, as reviewed by A. Bos-Mikich et al.

Last but not least, the microRNAs, noncoding RNA molecules mediating the translational suppression and posttranscriptional control of gene expression, have been exposed by I. Virant-Klun et al. as extremely important regulators of developmental processes, pluripotency, differentiation, and also shift to cancer.

The manuscripts published in this special issue bring an important new knowledge to the field and arise some new interests and challenges for the future.

*Irma Virant-Klun* *Irma Virant-Klun* *Hong-Thuy Bui* *Hong-Thuy Bui* *Mariusz Z. Ratajczak* *Mariusz Z. Ratajczak*

